# A retrospective cohort study of pediatric hospitalization due to dentoalveolar infection before and after a change in national health insurance

**DOI:** 10.1038/s41598-022-25045-0

**Published:** 2022-11-28

**Authors:** Itai Zeevi, Sahar Abdulqader, Uri Zilberman, Moti Moskovitz, Avia Fux-Noy

**Affiliations:** 1 Faculty of Dental Medicine, Hebrew University of Jerusalem, P.O. Box 12272, 9112102 Jerusalem, Israel; 2 Department of Oral and Maxillofacial Surgery, Hadassah Medical Center, P.O. Box 12272, 9112102 Jerusalem, Israel; 3 Pediatric Dental Unit, Barzilai Medical Center, Hahistadrut 2, 78278 Ashkelon, Israel; 4 Department of Pediatric Dentistry, Hadassah Medical Center, P.O. Box 12272, 9112102 Jerusalem, Israel

**Keywords:** Health care, Medical research

## Abstract

This retrospective cohort study aimed to examine trends in pediatric (0–18 years old) hospitalizations due to dentoalveolar infection, before and after the inclusion of pediatric dental care in Israel’s National Health Insurance Law. Data were collected from the medical records of one oral and maxillofacial surgery department. Data were compared between patients hospitalized during 2002–2010 (group A, n = 531) and 2011–2019 (group B, n = 381). The mean age of the cohort was 8 years. A dentoalveolar abscess was the main cause of hospitalizations in both groups. Group B exhibited a higher rate of previous dental treatment in general (p = 0.001), and of previous dental treatment for the tooth responsible for the infection (p = 0.03). The prevalent treatment during hospitalization combined intravenous antibiotics and extraction, with or without drainage (58.1%) for group A; and intravenous antibiotics and drainage (49.4%) for group B (p < 0.01). Dental care provided by the Israel’s National Health Insurance should focus not only on operative treatment but also on oral health promotion and caries prevention, to reduce hospitalizations due to dentoalveolar infections.

## Introduction

An odontogenic infection develops from a tooth, usually following severe caries, trauma, deep restorations, or failed root canal treatment, and can be initiated by a variety of bacteria. A dental or dentoalveolar abscess denotes a localized collection of pus in the alveolar bone, at the root furcation or apex of the tooth. After entering the periapical tissues, these bacteria can induce acute inflammation, leading to pus formation^1,2^. Most odontogenic infections penetrate the bone such that they become vestibular abscesses. These infections are usually self-limiting^1,3^. The main symptoms of superficial dental infections include localized pain, swelling, cellulitis, tooth sensitivity to percussion, and erythema. Sometimes malaise and fever develop. However, if the primary source of the infection is not eliminated, the inflammation process can progress and spread into deep fascial spaces. This can result in more severe infections and present as swelling and fever, and sometimes as difficulty in swallowing, opening the mouth, or breathing. Multiple severe complications of odontogenic infections have been reported, such as airway obstruction, cavernous sinus thrombosis, mediastinitis, necrotizing fasciitis, sepsis, thoracic empyema, cerebral abscess, and osteomyelitis^1–3^.

Despite medical progress and intensive health education, odontogenic infections leading to surgical intervention and hospitalization are still common in children and adolescents^3–5^. According to nationwide data, 215,073 visits to emergency rooms due to dental conditions occurred in the United States in 2008, among patients aged 21 years and younger^4^. A single-center analysis conducted in Berlin reported 120 hospitalizations due to odontogenic abscesses during 2013–2015 in patients aged 1–17 years^5^. A study conducted by an Israeli healthcare provider with 2.3 million members, reported 1413 hospitalizations due to odontogenic infections in children younger than 18 years during 2005–2011^6^. Reporting this common problem is important for enabling epidemiological analysis, due to the implications on the healthcare system. Severe dental caries have been linked with considerably increased risks of hospitalization; the consequences include financial costs, loss of manhours, and substantial negative impact on economic productivity^7^.

In June 2010, the Israeli government approved including dental treatment for children in the National Health Insurance Law (NHIL)^8^. Initially, this included children from birth to age 8 years. The eligibility age was updated to 12 years in 2013; subsequently extended gradually to age 18 years^9^. In Israel, all citizens are required to choose one of four competing health plans for their health insurance. The health plans provide a broad package of benefits stipulated by the government. When pediatric dental care was included in the NHIL, the health plans invited their insurees to receive treatment at their public dental clinics. The treatment includes all preventive and operative procedures in pediatric dentistry. With this in mind, the aims of the present study were (1) to examine trends in pediatric hospitalizations due to dentoalveolar infection, following the inclusion of dental care for children in the NHIL, (2) to study the factors and patient characteristics associated with hospitalizations before and after inclusion of dental care for children in the NHIL. The null hypothesis was that the inclusion of dental care for children in the NHIL would not affect the number of hospitalized children every year.

## Methods

### Design and study group

This is a retrospective study of computerized medical records of patients who were hospitalized at the Oral and Maxillofacial Surgery Department at Hadassah Medical Center, Jerusalem, Israel.

The inclusion criteria were age 0–18 years and hospitalization due to dentoalveolar infection, in the years 2002–2019. The patients were divided into two groups according to the period of hospitalization: Group A comprised the patients who were hospitalized during 2002–2010, before the inclusion of pediatric dental care in the NHIL. Group B comprised the patients who were hospitalized during 2011–2019, after the inclusion of dental care in the NHIL.

Data collected from the patients’ medical records included demographic data such as age and gender. Other relevant data were collected as follows:

1. Dental history of the patient—whether the patient had ever visited a dentist before hospitalization (yes/no), and whether the tooth that was the source of infection was previously treated (yes/no).
2. Diagnosis at hospitalization—dental abscess or cellulitis.
3. The agent that referred to the emergency room—parent, dentist, or physician.
4. The tooth that was the source of infection—primary tooth, permanent tooth, or both.
5. Duration of the hospitalization, in days.
6. Antibiotic treatment prior to hospitalization—Penicillin (Amoxicillin), Augmentin (Amoxicillin and Clavulanate), other, or none.
7. Medical treatment provided at the hospitalization—tooth extraction, abscess drainage, intravenous (IV) antibiotics.

### Statistical analysis

IBM SPSS 22.0 software was used for statistical analysis, and p < 0.05 was considered a statistically significant difference. Descriptive statistics were presented as absolute numbers and percentages for the categorical variables; and as means, medians, and standard deviations (SD) for the continuous variables. Comparative statistics between the two groups were performed using the Chi-Square test, T-test, and Fisher’ exact test.

### Ethics approval and consent to participate

This study was performed in line with the principles of the Declaration of Helsinki. Approval was granted by the committee on research involving human subject of the Hebrew University- Hadassah Medical School, Jerusalem, Israel (Date: October 7th 2019/No. HMO-0507-19). Informed consent was waived off by the institutional ethics committee.

## Results

Of the 912 patients included, 531 were in group A, and 381 were in group B. Calculating the mean number of hospitalizations per year for the 9-year periods before and after the inclusion of pediatric dental care in the NHIL yielded 59 and 42 for the respective groups. The median age of both groups was 7 years; the mean age was 8 years (SD = 4 years). Both groups comprised a slightly higher proportion of boys than girls, without statistical significance (p = 0.387). Patient and hospitalization features are listed in Table 1.

**Table 1:** Patient and hospitalization characteristics before and after (group A and B, respectively) inclusion of pediatric dental care in Israel’s National Health Insurance Law.

		Group A (2002–2010) N (%)	Group B (2011–2019) N (%)	All N (%)	p-value
Total number		531	381	912	
Gender	M	301 (56.7)	226 (59.3)	527 (57.8)	0.387
	F	230 (43.3)	155 (40.7)	385 (42.2)	
Previous dental care—general	Yes	130 (24.5)	131 (34.4)	261 (28.6)	0.001*
	No	401 (75.5)	250 (65.6)	651 (71.4)	
Previous dental care—infected tooth	Yes	106 (20.0)	100 (26.3)	206 (22.6)	0.03*
	No	425 (80.0)	281 (73.7)	706 (77.4)	
Diagnosis	Abscess	511 (96.2)	373 (97.9)	884 (96.9)	0.176
	Cellulitis	20 (3.8)	8 (2.1)	28 (3.1)	
Origin tooth	Primary	373 (70.2)	260 (68.2)	633 (69.4)	0.56
	Permanent	136 (25.6)	108 (28.4)	244 (26.8)	0.364
	Both	22 (4.2)	13 (3.4)	35 (3.8)	0.605
Sextant	1 (upper right)	93 (17.5)	75 (19.7)	168 (18.4)	0.436
	2 (upper front)	73 (13.8)	45 (11.8)	118 (12.9)	0.44
	3 (upper left)	111 (20.9)	77 (20.2)	188 (20.6)	0.868
	4 (lower left)	126 (23.7)	81 (21.3)	207 (22.7)	0.423
	5 (lower front)	9 (9)	6 (1.6)	15 (1.6)	1
	6 (lower right)	119 (22.4)	97 (25.5)	216 (23.9)	0.305
Treatment	IV ant. + Drainage	124 (23.4)	188 (49.4)	312 (34.2)	< 0.001*
	IV ant. + extr. +/− drain.	309 (58.1)	180 (47.2)	489 (53.6)	< 0.01*
	IV ant. only	98 (18.5)	13 (3.4)	111 (12.2)	< 0.001*
Prior antibiotics	Moxypen	168 (31.6)	127 (33.3)	295 (32.4)	0.616
	Augmentin	162 (30.5)	162 (42.5)	324 (35.5)	< 0.001*
	Other	63 (11.9)	27 (7.1)	90 (9.9)	*0.018
	Nothing	138 (26)	65 (17.1)	203 (22.3)	*0.0016
Referral agent	Parent	60 (11.3)	158 (41.5)	218 (23.9)	< 0.001*
	Physician	44 (8.3)	68 (17.9)	112 (12.3)	< 0.001*
	Dentist	45 (8.5)	76 (20.0)	121 (13.3)	< 0.001*
	Unknown	382 (71.9)	79 (20.7)	461 (50.6)	< 0.001*
Referral agent, calculated from the known data**	Parent	60 (40.3)	158 (52.3)		0.155
	Physician	44 (29.5)	68 (22.5)		0.222
	Dentist	45 (30.2)	76 (25.2)		0.389

*ant.* Antibiotics, *extr.* Extraction, *drain.* drainage.

*p-value < 0.05.

**Data were missing of 382 (71.9%) of group A and 79 (20.7%) of group B.

Significant values are in bold.

A dentoalveolar abscess was the main reason for hospitalization in the two study groups: 511 patients (96.2%) in group A, and 373 patients (97.9%) in group B.

**Figure 1:**
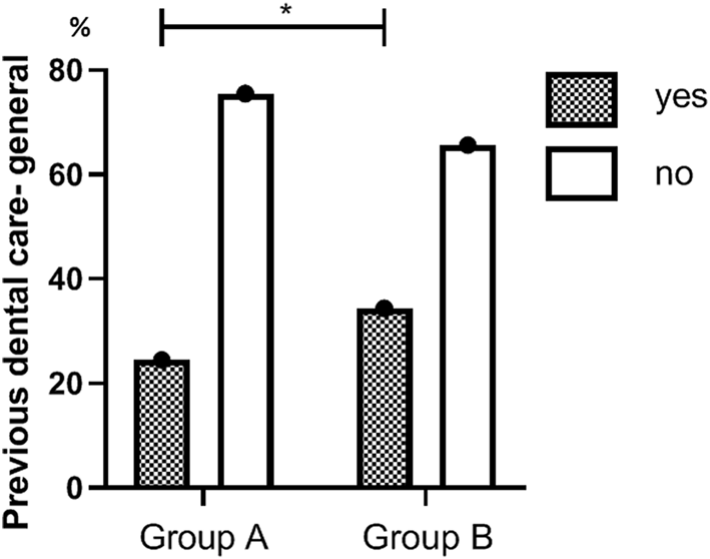
Previous dental care of patients hospitalized before and after (group A and B, respectively) inclusion of pediatric dental care in Israel’s National Health Insurance Law. The bars show the percentages of patients who received dental care prior to hospitalization. The difference between the groups in the percentage of those who received treatment is statistically significant. *p-value < 0.05.

**Figure 2:**
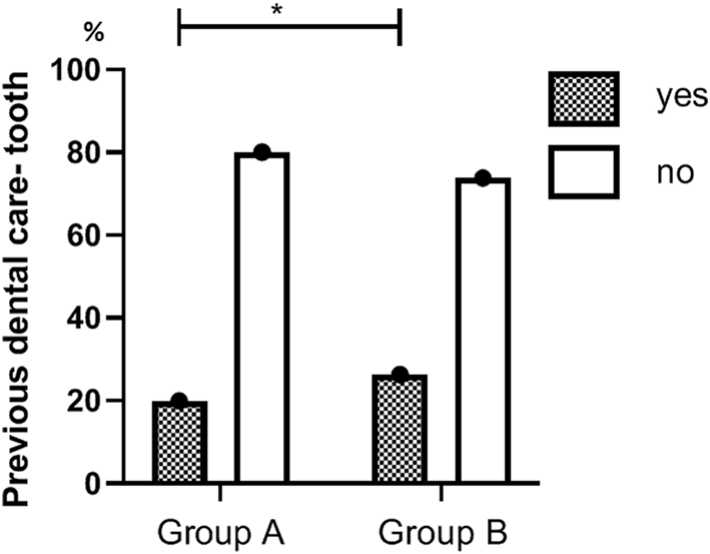
Previous dental care of the infected tooth, among patients hospitalized before and after (group A and B, respectively) inclusion of pediatric dental care in Israel’s National Health Insurance Law. The bars show the percentages of patients who received dental care prior to hospitalization, for the tooth that was the source of the infection. The difference between the groups in the percentage of those who received treatment is statistically significant. *p-value < 0.05.

For group A compared to group B, prior dental treatment was recorded for a lower proportion of patients: 24.5% (130) versus 34.4% (131), p = 0.001 (Fig. 1). For these respective groups, previous dental history of the tooth that prompted the hospitalization was recorded for a lower proportion of patients: 20.0% (106) versus 26.3% (100), p = 0.03 (Fig. 2). A primary tooth was the most common origin of infection in both groups: 70.2% and 68.2%, respectively.

**Figure 3:**
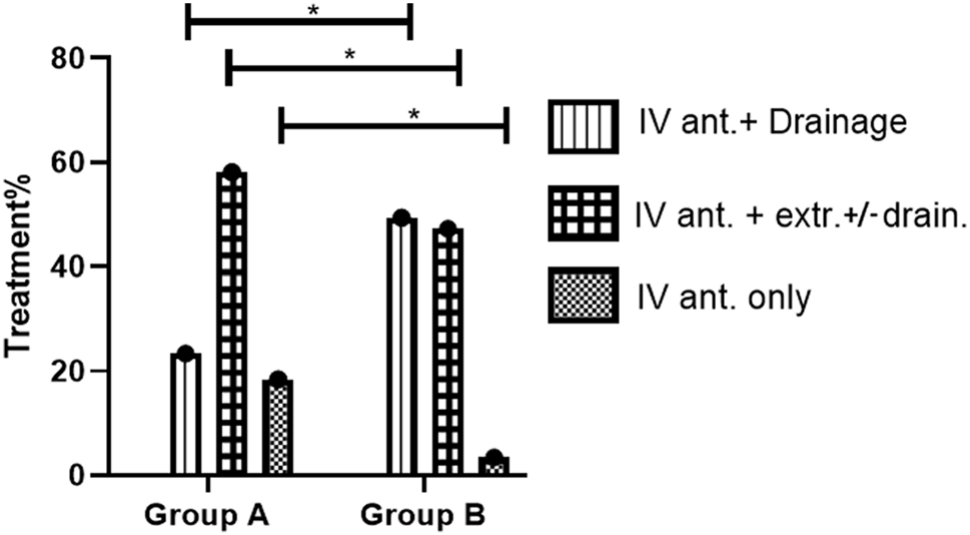
Medical treatment administered during hospitalization among patients hospitalized before and after (group A and B, respectively) inclusion of pediatric dental care in Israel’s National Health Insurance Law. *IV ant.* intravenous antibiotic, *extr.* Extraction, *drain.* drainage. *p-value < 0.05.

The treatment provided to all hospitalized patients included the administration of IV antibiotic. For some patients, the treatment also included extraction of the tooth involved, drainage of the abscess, or a combination of the two. Among group A patients, the therapeutic approach was most often combined IV antibiotics and tooth extraction, with or without drainage (58.1%). Among group B patients, the therapeutic approach most often combined IV antibiotic and abscess drainage (49.4%). This difference was statistically significant (p < 0. 01) (Fig. 3).

**Figure 4:**
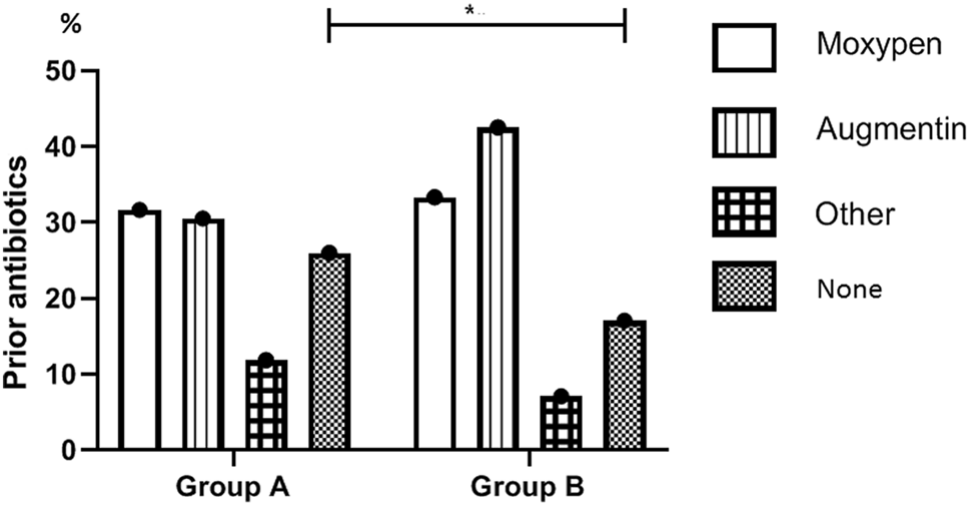
Antibiotics administered prior to hospitalization among patients hospitalized before and after (group A and B, respectively) inclusion of pediatric dental care in Israel’s National Health Insurance Law. *p-value < 0.05.

Most patients received antibiotics to relieve signs of inflammation before arriving at the hospital. While Moxypen (Amoxicillin) was the most common antibiotic administered to group A patients (31.5%), Augmentin (Amoxicillin and Clavulanate) was most commonly administered to group B patients (42.5%). Additionally, 138 (26.0%) of the patients in group A did not receive any antibiotics prior to arriving at the hospital, compared to 65 patients (17.0%) in group B; this difference was statistically significant (p < 0.01) (Fig. 4).

The referring agent was not specified in the medical records of the majority of patients (71.9%) of group A, and was unknown in only 79 (20.7%) of group B (Table 1). In an analysis that included only patients for whom the referring agent was known, this parameter did not differ between the groups.

For the two groups, the mean and median days of hospitalization were the same, 4 (Table 2). However, 163 (30.0%) group A patients were hospitalized for up to 3 days; and the rest were hospitalized for 4 days or more. This compares to the hospitalization of 183 (48%) group B patients for 3 days, and the rest for 4 days or more. This difference was statistically significant (p < 0.001).

**Table 2:** Hospitalization duration (days) before and after (group A and B, respectively) inclusion of pediatric dental care in Israel’s National Health Insurance Law.

		Group A (2002–2010)	Group B (2011–2019)	All	p-value
Range		2–19	1–84	1–84	
Mean (SD)		4 (4)	4 (4)	4 (4)	
Median		4	4	4	
Hospitalization duration N (%)	1	0 (0)	6 (1.6)	6 (0.7)	1
	2	22 (4.2)	46 (12.1)	68 (7.5)	0.052
	3	141 (26.6)	131 (34.4)	272 (29.8)	< 0.001*
	4	182 (34.3)	110 (28.9)	292 (32.0)	< 0.001*
	5	102 (19.2)	44 (11.6)	146 (16.0)	< 0.001*
	6	45 (8.5)	19 (5.0)	64 (7.0)	0.082
	More than 6	39 (7.3)	25 (6.6)	64 (7.0)	0.695

*p-value < 0.05.

Significant values are in bold.

## Discussion

The main finding of this study was that after the inclusion of dental care for children in the NHIL, the number of children hospitalized every year decreased. Thus, the null hypothesis was rejected. Notably, most of the hospitalized children in both time periods were those who did not receive any previous dental treatment.

We report a lower annual number of patients admitted to the hospital due to dentoalveolar infection in the period after dental treatments were included in the NHIL. Possibly, the inclusion of dental care in health services led to an increase in dental care and therefore reduced the number of hospitalizations. The hypothesis that the inclusion of dental care in insurance coverage can lead to decreased hospitalizations due to dental infection is supported by the research of Morgan et al.^10^. They reported that the inclusion of dental care in insurance coverage in the U.S. was followed, in 2010–2017, by decreased numbers of emergency room visits and hospitalizations due to dental infection, of individuals aged 0–20 years. Decreased hospitalizations were observed among children with access to dental care compared to uninsured children of low socioeconomic status. Bowe et al.^11^ reported an increase in the number and severity of dentoalveolar infections in patients aged 15–91 years during a period when public health care dental benefits were restricted. However, the inclusion of dental care cannot be concluded as the only reason for the decrease in the number of hospitalizations, as other factors may have changed between the periods. Changes in population size, the total number of hospitalizations, and hospitalizations in other hospitals in the study area were not examined. Notably, Hadassah Medical Center is a university hospital and services are provided under NHIL. However, dental care, without hospitalization facilities, is also provided by several public and private clinics in the area. In 2014, an additional department of oral and maxillofacial surgery was established in another public hospital in the study area, and this could have affected the results.

The Israel Health Policy Research Institute^12^ found that in 2016, after the inclusion of dental treatment in the NHIL for children up to age 12 years, 64% of children had visited a dentist during the preceding year, and 25% of the children had never visited a dentist. The most common reason for visiting a dentist was a periodic checkup initiated by the parents (47%); emergency visits (22%) were the next most common reason. This indicates that, even when dental care was provided without charge, a substantial number of children still did not regularly visit dental clinics^12^. Efforts should be made to encourage routine periodic visits of all children to the dental clinic. The barriers to reaching the clinic were not examined in this study, but presumably, geographical location and availability of appointments influence attendance. Expanding dental health reform and addressing barriers to preventive dental care, especially among minorities and those of lower educational level, may help reduce households’ private expenses on dental care^13^.

We report a higher proportion of patients with previous dental visits or with a history of dental care in the infected tooth, in the years after the inclusion of dental care in the NHIL, compared to the preceding years. Presumably, more children received dental care; therefore, the dental history of more children who had been hospitalized included dental care. This corroborates the study by Natapov et al.^14^. They reported increased administration of dental care to children after the introduction of dental care in the NHIL. On the other hand, the data collected in this study did not include the type of treatment given. The treatment may have included only temporary measures for pain relief, and not completion of definitive dental treatment. It is also possible that the treatments given contributed to complications and the development of odontogenic infections. According to Chisini et al.^15^, the main reason for restoration failure in primary teeth was secondary caries (36.5%). Class I restorations and restorations placed using a rubber dam presented better success rates. The high variation in materials used may be due to children’s behavior, which affects the duration of the appointment and control of the environment, and thus the quality of the procedure. Moskovitz et al.^16^ found that 3.3% of primary molars that underwent pulpectomy treatment developed a new periapical radiolucency, or an extension of the existing radiolucent lesion. Sandhyarani et al.^17^ reported the development of dentoalveolar and radicular cysts in primary molars following pulpectomy treatment with zinc oxide eugenol. They also stressed the importance of regular follow-up, to improve the quality of dental care performed. Indeed, electronic devices are reliable means of obtaining working length in primary molars and have been recommended for clinical implementation of endodontics in primary teeth^18^. Nonetheless, the complex and branched anatomy of primary root canals makes it difficult to disinfect mechanically and chemically, rendering variable prognosis^19^. In addition, the success of the treatment depends on the materials used for pulp treatments, which show varying success rates^20^. This does not imply that pulpectomy treatments can lead to hospitalizations; pulp treatment for extensive decay in primary teeth is generally successful^20^ and root canal treatment has been shown to be successful even with abscessed primary teeth^21^.

Fewer extractions were performed in the later than the earlier period. The difference in treatment protocol may reflect a change in the therapeutic approach implemented in the Oral and Maxillofacial Surgery Department during the two-decade study period, to a more conservative approach. Changes in the department’s personnel, including the integration of younger senior dentists, may be reasons for changes in dental practice. This treatment approach is in accordance with Goncalves et al.^22^, who found that the most effective treatment for a dentoalveolar infection is a combination of IV antibiotic and drainage.

A larger proportion of patients in the earlier than the later period was hospitalized for 4 days and more. In addition, a larger proportion of patients in the earlier than the later period did not receive any antibiotic treatment prior to arriving at the hospital. These two sets of results could be connected. It is possible that the lack of antibiotic treatment before hospitalization led to a more serious medical condition, which required a longer hospitalization. Additionally, the hospitalization period may have been influenced by the therapeutic approach that was taken. In the later compared to the earlier period, a smaller proportion of patients received IV antibiotics alone, and a larger proportion underwent surgical treatment (drainage with or without tooth extraction) in addition to IV antibiotics.

Of note, several patients were hospitalized for a prolonged period, over 30 days. The long hospitalizations were probably due to other background diseases; however, retroactive recovery of the duration of treatment from the medical records of the Department of Oral and Maxillofacial Surgery is not possible. Such data may have influenced the results, but as this is relevant to only isolated patients, the overall effect is probably negligible.

As the referring agent of most patients in the earlier period was unknown, conclusions cannot be drawn regarding this information. Increasing accessibility of dental care for children increases the chances that the first person contacted in the case of an emergency will be a dentist, and that this professional will refer the patient to the emergency room when necessary. Therefore, an increase in referrals from dentists compared to self-referrals and physician referrals may be expected.

Limitations of the study and future research: The retrospective design did not enable attaining the full details and characteristics related to the hospitalizations. This information includes the socioeconomic background of the patients and the reasons for attending past dental treatments (routine treatments or emergency only), the type of facility (public or private) where previous treatment was received, the time elapsed from the last dental treatment to hospitalization, and the duration and number of times of antibiotic treatment before hospitalization. A bias arises that is inherent to “before and after studies”. Namely, factors other than the change in the NHIL may have changed between the periods and influenced the results. These factors include dental treatment approaches, such as the use of pulp therapy versus extractions; and the dental materials used for pulp therapy and restorations. Future studies are needed to fully understand the influence of the inclusion of dental care in NHIL on hospitalization rates.

Dental care provided in the NHIL should be of high quality. This includes a high availability of dentists and appointments, correct diagnosis, the use of updated dental materials, and the performance of dental treatments with high success rates. Moreover, dental care should focus not only on operative treatment but also on oral health promotion and caries prevention, to reduce hospitalizations due to dentoalveolar infections.

## Data Availability

The datasets used and/or analyzed during the current study available from the corresponding author on reasonable request.

## References

[1] Ogle OE (2017). Odontogenic infections. Dent. Clin. N. Am..

[2] Shweta Prakash SK (2013). Dental abscess: A microbiological review. Dent. Res. J..

[3] Bali RK, Sharma P, Gaba S, Kaur A, Ghanghas P (2015). A review of complications of odontogenic infections. Natl. J. Maxillofac. Surg..

[4] Allareddy V (2014). Hospital-based emergency department visits with dental conditions among children in the United States: Nationwide epidemiological data. Pediatr. Dent..

[5] Doll C (2018). Odontogenic abscess-related emergency hospital admissions: A retrospective data analysis of 120 children and young people requiring surgical drainage. Biomed. Res. Int..

[6] Klivitsky A, Tasher D, Stein M, Gavron E, Somekh E (2015). Hospitalizations for dental infections: Optimally versus nonoptimally fluoridated areas in Israel. J. Am. Dent. Assoc..

[7] Pitts NB (2017). Dental caries. Nat. Rev. Dis. Primers.

[8] State of Israel - Ministry of Health, Division of Health Affairs. *Dental Services for Children in the National Health Insurance Law*. https://www.health.gov.il/hozer/mr20_2010.pdf (Accessed 12 April 2022) (2010).

[9] Israel Government Website. https://www.gov.il/he/service/dental-treatments-for-children (Accessed 12 April 2022).

[10] Morgan T (2021). National trends and characteristics in emergency department visits for nontraumatic dental conditions among pediatric patients. Pediatr. Dent..

[11] Bowe CM, Gargan ML, Kearns GJ, Stassen LF (2015). Does access to general dental treatment affect the number and complexity of patients presenting to the acute hospital service with severe dentofacial infections?. J. Ir. Dent. Assoc..

[12] Ashkenazi, Y., Zusman, S. & Natapov, L. *Patterns of Utilization and Experiences of Children in Dental Care Following the Reform in Dental Care in Israel*. http://brookdaleheb.jdc.org.il/_Uploads/dbsAttachedFiles/dentist-1.pdf (Accessed 17 May 2022) (Myers JDC Brookdale Institute, 2014).

[13] Orenstein L, Chetrit A, Oberman B, Benderly M, Kalter-Leibovici O (2020). Factors associated with disparities in out-of-pocket expenditure on dental care: Results from two cross-sectional national surveys. Isr. J. Health Policy Res..

[14] Natapov L, Sasson A, Zusman SP (2016). Does dental health of 6-year-olds reflect the reflect the reform of the Israeli system?. Isr. J. Health Policy Res..

[15] Chisini LA (2018). Restorations in primary teeth: A systematic review on survival and reasons for failures. Int. J. Paediatr. Dent..

[16] Moskovitz M, Yahav D, Tickotsky N, Holan G (2010). Long-term follow up of root canal treated primary molars. Int. J. Paediatr. Dent..

[17] Sandhyarani B, Noorani H, Shivaprakash PK, Dayanand AH (2016). Fate of pulpectomized deciduous teeth: Bilateral odontogenic cyst?. Contemp. Clin. Dent..

[18] Kielbassa AM, Muller U, Munz I, Monting JS (2003). Clinical evaluation of the measuring accuracy of ROOT ZX in primary teeth. Oral Surg. Oral Med. Oral Pathol. Oral Radiol. Endod..

[19] Ahmed HM (2013). Anatomical challenges, electronic working length determination and current developments in root canal preparation of primary molar teeth. Int. Endod. J..

[20] Smaïl-Faugeron V (2018). Pulp treatment for extensive decay in primary teeth. Cochrane Database Syst. Rev..

[21] Kielbassa AM, Attin T, Schaller HG, Hellwig E (1995). Endodontic therapy in a postirradiated child: Review of the literature and report of a case. Quintessence Int..

[22] Gonçalves L, Lauriti L, Yamamoto MK, Luz JG (2013). Characteristics and management of patients requiring hospitalization for treatment of odontogenic infections. J. Craniofac. Surg..

